# Participation motivation and athletes’ competitive anxiety, mediating role of psychological resilience and moderating role of sport commitment

**DOI:** 10.3389/fpsyg.2026.1824267

**Published:** 2026-08-20

**Authors:** Lifu He, Ying Liu

**Affiliations:** School of Physical Education, Zhengzhou Normal University, Zhengzhou, Henan, China

**Keywords:** competitive anxiety, participation motivation, psychological resilience, sport commitment, university student athletes

## Abstract

**Background:**

As competitive demands in sport increase, competitive anxiety has become an important concern for athletes. Such anxiety may interfere with athletes’ performance during a specific competitive event and may also be associated with longer-term mental health difficulties. While existing research has focused on athletes’ competitive anxiety, the understanding of its underlying psychological mechanisms remains insufficient. Based on the conservation of resources theory, this study intends to explore the relationship between participation motivation and athletes’ competitive anxiety, with an emphasis on the mediating role of psychological resilience and the moderating role of sport commitment.

**Methods:**

This study used a questionnaire survey to gather information from 711 Chinese university student athletes. The variables of participation motivation, psychological resilience, sport commitment, and competitive anxiety were measured. Structural Equation Modeling was used to analyze the data.

**Results:**

The findings showed a significant negative link between participation motivation and competitive anxiety, with psychological resilience acting as a key mediating factor. Sport commitment significantly moderated the link between participation motivation and competitive anxiety.

**Conclusion:**

This study reveals the mechanisms through which participation motivation, psychological resilience, and sport commitment are associated with competitive anxiety among university student athletes. The results suggest that psychological resilience and sport commitment help reduce competitive anxiety, providing both theoretical foundations and practical guidance for psychological interventions aimed at university student athletes.

## 1 Introduction

Competitive anxiety is an immediate state anxiety response that athletes experience in a specific competition because of concerns about their performance, the competition outcome, or external evaluation (Chen et al., 2024). Excessive competitive anxiety may be associated with difficulty maintaining attention, greater concern about the risk of failure, and a reduced ability to fully use existing skills during competition (Li et al., 2023; Peng and Zhang, 2021). Competitive anxiety often varies across competition stages and situational demands. A single episode does not necessarily indicate long-term psychological problems. However, when competitive anxiety recurs during high-load training, preparation for important events, or consecutive competitions without sufficient recovery, continued resource depletion may be associated with emotional exhaustion, sport burnout, and mental health difficulties. It may also limit sustained engagement in training and long-term athletic development (Tóth et al., 2022; Zhou et al., 2024). Therefore, competitive anxiety needs to be understood from the perspective of how psychological resources are acquired, maintained, and depleted.

Compared with professional athletes, university student-athletes have a distinct social and developmental background. They are transitioning from university life to adulthood and career development while managing the dual roles of student and athlete. They need to complete academic tasks, such as coursework and examinations, and also participate in long-term training and sports competitions (Chen and Peng, 2025; Fuentes-García et al., 2020). They also face multiple expectations from coaches, teammates, families, and universities. Previous studies have shown that these performance expectations may serve as important sources of stress and are associated with higher competitive anxiety (Berastegui-Martínez et al., 2026; Endo et al., 2023). When university student-athletes perceive these expectations as excessive, conflicting, or beyond their available time and psychological resources, they may experience greater tension, worry, and emotional fluctuations as competitions approach (Mendoza Balite et al., 2021; Peng and Zhang, 2021). However, existing research has paid limited attention to how university student-athletes mobilize psychological resources to cope with competitive pressure and to the related psychological mechanisms and boundary conditions.

Participation motivation, psychological resilience, and sport commitment may offer important perspectives for understanding competitive anxiety among university student-athletes. Prior research has usually examined the links of these variables with competitive anxiety separately. Few studies have analyzed their interrelationships within a unified theoretical framework. In particular, whether participation motivation is indirectly associated with lower competitive anxiety through psychological resilience and whether sport commitment changes the strength of these associations have not been fully examined. Therefore, based on conservation of resources (COR) theory, this study investigates the link between participation motivation and competitive anxiety among university student-athletes. Additionally, it tests the mediating role of psychological resilience and the moderating role of sport commitment.

### 1.1 Theoretical foundation

The COR hypothesis states that people work hard to get, preserve, and protect resources that help them achieve their goals and cope with stress. When important resources are endangered or lost, or when resource investment does not produce the anticipated returns, people are more prone to experience stress and negative emotional responses (Hobfoll, 1989). In contrast, adequate psychological and behavioral resources can help individuals appraise stressful situations, regulate their emotions, and maintain goal-directed behavior (Hobfoll, 1989; Tang et al., 2022). The theory also emphasizes that continued resource loss may create a loss spiral, making it increasingly harder for individuals to cope effectively with subsequent stressors.

In competitive settings, athletes must continuously invest attentional resources, emotion regulation capacity, and physical energy. Uncertainty about competition outcomes, external evaluation, and performance pressure may lead them to perceive threats to these resources. When their available resources appear insufficient to meet competition demands, or when failure may place already invested resources at risk, competitive anxiety may be higher (Tahir et al., 2024). Participation motivation may support athletes’ engagement in training, pursuit of goals, and positive experiences. It can therefore be understood as a motivational basis for resource investment and resource acquisition (Haase, 2021). Stronger participation motivation may be associated with greater focus on skill development and personal growth, as well as less excessive concern about failure and external evaluation. It may therefore be related to lower competitive anxiety (Bochaver et al., 2023). Psychological resilience reflects athletes’ capacity to remain adaptive under stress and setbacks, regulate negative emotions, and restore psychological resources. It may therefore be associated with lower competitive anxiety (Wang, 2021). Sport commitment reflects athletes’ tendency to maintain their goals and continue investing resources. Different levels of sport commitment may shape how athletes use participation motivation and psychological resources to cope with competition demands (Hu et al., 2023; Mendoza Balite et al., 2021). Therefore, this study develops a theoretical model based on three aspects: resource investment, resource protection and recovery, and the conditions for continued resource investment. It examines the link between participation motivation and competitive anxiety, the mediating role of psychological resilience, and the moderating role of sport commitment.

### 1.2 Participation motivation and competitive anxiety

Extrinsic and intrinsic motivation are the two categories into which psychological study separates motivation. Our research focuses on the psychological drive people have when playing sports because of their own interests or needs, which is known as intrinsic participation motivation. The scale used in this study includes five elements: health motivation, appearance motivation, hedonic motivation, social motivation, and competence motivation. These dimensions encompass the various reasons individuals engaging in physical activities (Guo et al., 2024). Studies have indicated a significant negative link between participation motivation and competitive anxiety (Tahir et al., 2024). Reyes-Hernández et al. (2021) discovered that higher participation motivation effectively reduces individuals’ competitive anxiety. High levels of participation motivation typically indicate that athletes are passionate about their sport and view it as a means of personal growth and challenge. This allows them to focus more on the process rather than the outcome, reducing the fear of failure and excessive concern about external evaluation, thereby effectively alleviating competitive anxiety (Reyes-Hernández et al., 2021). Conversely, athletes with insufficient participation motivation are more likely to focus more on external outcomes and evaluations, experiencing a strong fear of failure. They are more likely to feel tense in intense competition and high-pressure environments, which exacerbates competitive anxiety (Haase, 2021; Pineda-Espejel et al., 2021). In addition, stronger participation motivation may increase the personal relevance of the competition, thereby mitigating competitive anxiety. From the standpoint of COR theory, participation motivation may be associated with greater training engagement, stronger goal persistence, and the accumulation of psychological resources. These resources may help athletes cope with competition pressure and may be related to lower competitive anxiety (Hobfoll, 1989; Tahir et al., 2024). Therefore, enhancing athletes’ participation motivation is essential for alleviating their competitive anxiety.

### 1.3 The mediating role of psychological resilience

Psychological resilience is the capacity to positively adapt and recover when facing adversity, pressure, or challenges in sports competition, as well as the ability to maintain mental health when encountering significant difficulties. It entails having the capacity to overcome obstacles, stay focused, and demonstrate perseverance in the face of challenges (Li and Pan, 2025). Research has shown a negative link between resilience and competitive anxiety (González-Hernández et al., 2020). Athletes with greater psychological resilience can stay calm during competitions, focusing on personal growth rather than excessively worrying about failure or external evaluation. This enables them to cope competitive pressure more effectively and reduce competitive anxiety (Yu et al., 2024). Psychological resilience also enhances athletes’ confidence, helping them maintain lower levels of anxiety when facing pressure and uncertainty during competitions (Wang, 2021). Zhang et al. (2023) discovered that athletes with higher psychological resilience generally exhibit reduced competitive anxiety and achieve better performance in competitions. In contrast, athletes with low psychological resilience may experience self-doubt and are more susceptible to competitive pressure, resulting in higher levels of anxiety (Tang et al., 2023). Additionally, studies have shown a strong positive link between participation motivation and resilience (Manzano Sánchez, 2022). The stronger the motivation, the more athletes are able to demonstrate sustained effort and determination in the face of setbacks, thereby exhibiting greater psychological resilience (Bochaver et al., 2023; Leon-Guereno et al., 2020). Strong participation motivation not only enhances athletes’ goal-oriented abilities but also promotes self-regulation, helping them set clear goals and overcome difficulties when facing pressure, thereby boosting psychological resilience (Lau et al., 2024; Prayag et al., 2023). In competitive sports, athletes often face intense external evaluation and criticism. Strong participation motivation helps them better manage their emotions, stay positive when facing difficulties, and strengthen psychological resilience (Alexe et al., 2022; Chen et al., 2026). Therefore, psychological resilience helps athletes effectively cope with challenges and pressure, alleviating competitive anxiety (Tang et al., 2023; Yigit and Ozisli, 2026). Trigueros et al. (2020) discovered that intrinsic motivation can increase athletes’ psychological resilience and reduce their anxiety (Wang, 2021). In conclusion, psychological resilience could be a crucial mediator between participation motivation and competitive anxiety.

### 1.4 The moderating role of sport commitment

Sport commitment highlights the athlete’s willingness and determination to persist in participating in sports, emphasizing the athlete’s commitment to long-term involvement in sports (Zhang et al., 2024). Participation motivation is the psychological forces that forces an individual to play sports in the short term, driven by intrinsic needs (Guo et al., 2024). Although both constructs involve sports participation, they differ in their focus. Participation motivation is short-term and immediate, primarily explaining why individuals begin participating in sports, whereas sport commitment is a long-term investment, focusing on how individuals cope with challenges and maintain participation over time. Therefore, these two constructs are complementary and can explain athletes’ behavior from different perspectives. In this study, participation motivation sparks the initial drive for athletes’ involvement, while sport commitment helps athletes sustain long-term engagement when facing challenges.

Sport commitment moderates the relationship between participation motivation and competitive anxiety, primarily because it enhances athletes’ focus and goal orientation (Simon-Piqueras et al., 2023). High sport commitment athletes tend to align their participation motivation with personal goals, rather than focusing solely on short-term outcomes or external pressure (Tao and Yu, 2025). This sense of commitment allows athletes to view competitive pressure as a challenge instead of a threat, resulting in reduced competitive anxiety (Hu et al., 2023). Specifically, high levels of sport commitment make athletes more focused on self-improvement and skill development, rather than excessively worrying about external evaluation or competition results (Guo et al., 2024; Joung et al., 2023). Therefore, sport commitment helps athletes reduce their perception of competitive anxiety by enhancing their positive response to participation motivation.

Given the lack of direct evidence that sport commitment moderates the other two paths, this study does not propose hypotheses about its moderating role in the link between participation motivation and psychological resilience or in the link between psychological resilience and competitive anxiety. Future research should further examine these possibilities.

### 1.5 Current research

**Figure 1:**
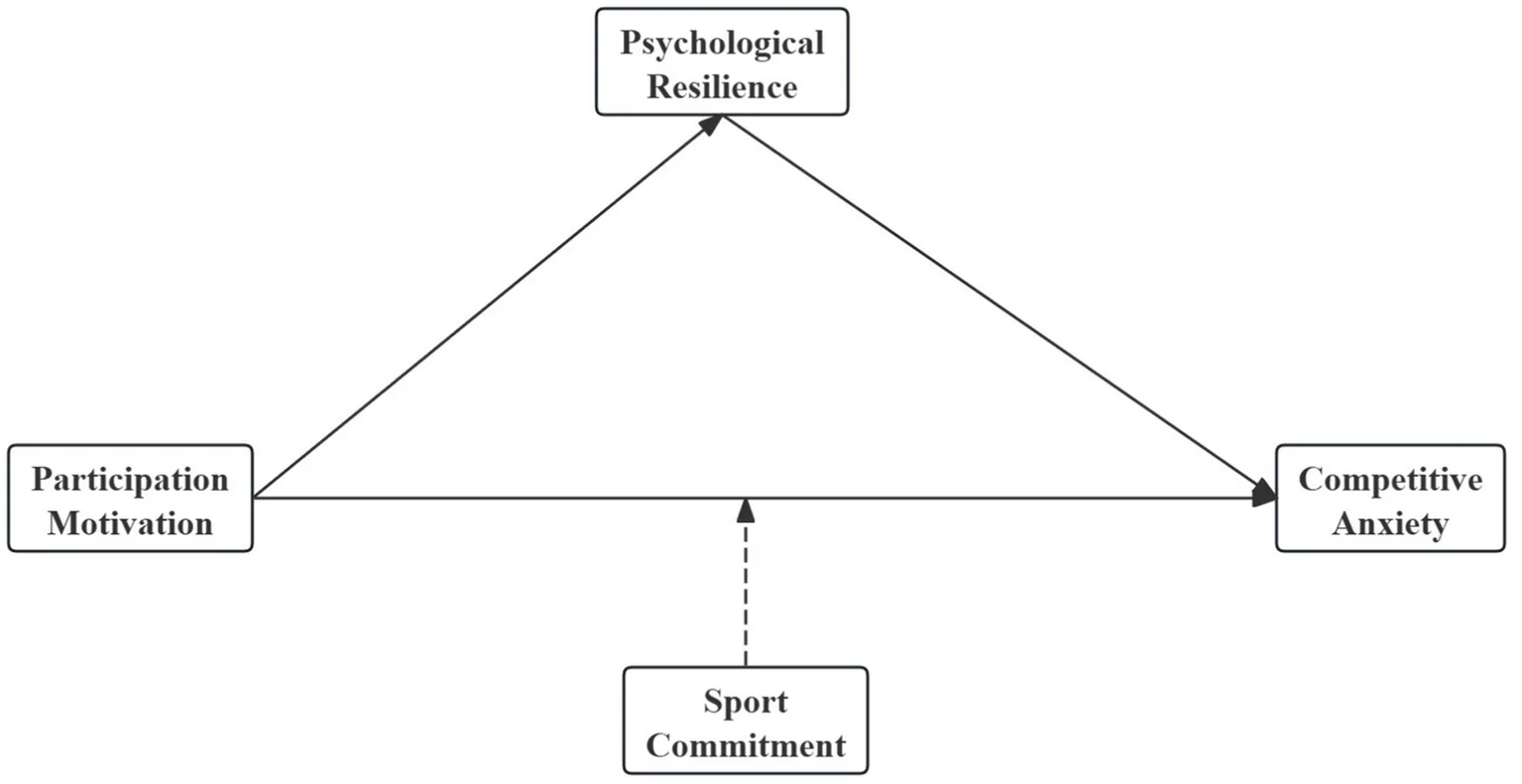
Research model.

Although existing studies have investigated competitive anxiety among athletes, its underlying psychological mechanisms have not been well examined. Currently, no research has explicitly revealed whether participation motivation is related to competitive anxiety in university student athletes through psychological resilience, nor has there been an exploration of whether sport commitment moderates the link between participation motivation and competitive anxiety. Therefore, based on COR theory, this study constructs a mediating model with a moderating effect. It aims to explore whether participation motivation is related to competitive anxiety in university student athletes, with psychological resilience serving as a mediator, while also examining the moderating effect of sport commitment in this process. This model is designed to explore the psychological mechanisms behind competitive anxiety among university student athletes, offering theoretical understanding and practical guidance for psychological interventions targeting this group. The conceptual model for this study is presented in Figure 1, from which the following research hypotheses are formulated:

*H1*: Participation motivation is strongly negatively related to competitive anxiety.

*H2*: Psychological resilience mediates the link between participation motivation and competitive anxiety.

*H3*: Sport commitment moderates the link between participation motivation and competitive anxiety.

## 2 Methods

### 2.1 Participants and procedure

To improve sample representativeness and reduce selection bias, this study used a simple random sampling approach to administer a questionnaire survey among university student athletes from three Chinese universities. The survey was administered and data was gathered between June to July in 2025. By contacting the university sports departments and sports colleges, the research team obtained lists of varsity team members and students majoring in sports-related fields, which provided the foundational data for subsequent sampling. Based on these lists, the research team randomly selected samples from athletes across different grades and majors. Before the formal survey, the research team thoroughly explained the study’s objective and process to both the universities and the students. The online questionnaire link, created using the Wenjuanxing^1^ platform, was then distributed through class group chats. Within the allotted time, students independently finished and turned in the questionnaire. According to Kline (2018), each measurement item should have at least 10 samples. The necessary sample size for this study, which has 56 items and a 20% sample attrition rate, was determined to be 672 participants (56 × 10 + 20% × 56 × 10). Seven hundred and thirty six surveys in all were gathered. Seven hundred eleven valid questionnaires were kept after 25 invalid responses (including those with more than 20% missing items or over 80% of items scored as “Strongly Agree” or “Strongly Disagree”; Meade and Craig, 2012; Wang et al., 2012) were eliminated, resulting in a 96.60% effective response rate. Participants were primarily distributed in their sophomore year (27.4%) (see Table 1). Before completing the questionnaire, all participants completed an informed permission form to guarantee that the study complied with ethical standards and protected participants’ privacy and autonomy.

**Table 1:** Descriptive statistics.

Constructs	Category	Quantity	Percentage (%)
Gender	Male	356	50.1
	Female	355	49.9
Grade	Freshman	188	26.4
	Sophomore	195	27.4
	Junior	193	27.1
	Senior	135	19.0
Training experience	0–1 Year	290	40.8
	2–3 Years	275	38.7
	4 Years or More	146	20.5

### 2.2 Measurement tools

#### 2.2.1 Participation motivation

This study used the Motivation for Physical Activity Measure, originally created by Ryan et al. (1997) and updated by Chen et al. (2013), to assess athletes’ participation motivation (Liu et al., 2023). The scale includes 15 items, covering five dimensions: health motivation, appearance motivation, enjoyment motivation, social motivation, and competence motivation. It uses a 5-point Likert scale. The mean score was calculated as the overall participation motivation score, with higher scores reflect greater participation motivation. The Cronbach’s *α* for the global score was 0.939.

#### 2.2.2 Psychological resilience

This study used the revised Connor-Davidson Resilience Scale by Connor and Davidson (2003) to assess athletes’ resilience (Mei et al., 2022). The scale contains 10 items and uses a 5-point Likert scale. The mean score was estimated, with better scores indicate higher resilience. Cronbach’s α was 0.926.

#### 2.2.3 Sport commitment

This study employed the Sport Commitment Scale to evaluate athletes’ willingness to participate in sports (Guo et al., 2024). The scale includes 4 items and uses a 5-point Likert scale. The mean score was estimated, with higher scores indicate stronger sport commitment. Cronbach’s α was 0.819.

#### 2.2.4 Competitive anxiety

This study employed the Competitive State Anxiety Inventory, revised by Zhu (1994) according to Chinese norms, to assess athletes’ competitive anxiety (Zhao et al., 2024). When completing the scale, participants recalled the most recent formal competition in which they had participated and reported how they actually felt before that competition began. Therefore, this study assessed state anxiety reported in relation to a specific competition rather than a general or stable tendency to experience anxiety. The scale contains 27 items, including three dimensions: cognitive state anxiety, somatic state anxiety, and state self-confidence. Items are rated on a 4-point Likert scale, and 10 items are reverse-scored. After reverse scoring, the mean score was estimated, with higher scores indicating greater competitive anxiety. Cronbach’s α was 0.955. The reason for choosing this scale is its ability to effectively capture athletes’ immediate anxiety responses in competitive or performance situations. Compared to trait competitive anxiety, state anxiety more accurately reflects an athlete’s psychological state at a specific moment, thereby helping us gain deeper insight into emotional fluctuations and psychological regulation processes in competitive settings.

### 2.3 Data analysis

This study used Smart PLS 4.0 for data analysis, employing the PLS-SEM method to explore the links between participation motivation, psychological resilience, sport commitment, and competitive anxiety. The study includes 711 valid questionnaires with four latent variables and 56 measurement items in the research model. After reverse-scoring the relevant items according to each scale’s scoring instructions, mean scores were calculated for descriptive statistics and correlation analysis. Higher values of the respective variables were indicated by higher scores. The use of item mean scores preserves the original range of the Likert scales and is a common scoring method in psychometric research. In the PLS-SEM analysis, all items were included as measurement indicators of their corresponding latent variables.

The statistical analyses followed a prespecified sequence. First, common method bias was assessed to investigate possible systematic bias resulting from same-source self-report data. Second, confirmatory factor analysis was conducted using AMOS 28 to examine the fit of the hypothesized measurement model. The degree of correspondence between the theoretical measurement model and the observed data was evaluated using chi-square to degrees of freedom ratio, comparative fit index, Tucker–Lewis index, incremental fit index, and root mean square error of approximation, thereby establishing an acceptable measurement basis for the subsequent structural model analysis. SmartPLS 4.0 was then used to evaluate indicator loadings, internal consistency reliability, convergent validity, and discriminant validity, thereby confirming the measurement quality of each latent variable. After the measurement model met the acceptable criteria, the research hypotheses were tested using a stepwise model-building approach. In the first step, a baseline model was established to investigate the direct association between participation motivation and competitive anxiety and the mediating role of psychological resilience. This step assessed the basic structural paths and the proposed indirect association. In the second step, an interaction term between participation motivation and sport commitment was added to the baseline model to test the moderating role of sport commitment. The interaction term was used to determine whether the association between participation motivation and competitive anxiety varied across levels of sport commitment. When the interaction effect was significant, simple slope analysis was conducted to interpret the direction and strength of this association at different levels of sport commitment. The mediation and moderation effects were not tested in two separate models. Instead, the relevant paths were added sequentially within the same theoretical framework. The significance of all direct, indirect, and interaction effects was assessed using 5,000 bootstrap resamples to obtain confidence intervals that relied less heavily on the assumption of normality.

## 3 Results

### 3.1 Descriptive statistics and correlation analysis

The correlation analysis indicated a significant positive link between participation motivation and psychological resilience (*r* = 0.64, *p* < 0.001), along with a significant negative link between participation motivation and competitive anxiety (*r* = −0.50, *p* < 0.001). Furthermore, resilience showed a strong negative correlation with competitive anxiety (*r* = −0.56, *p* < 0.001) (Table 2).

**Table 2:** Descriptive statistics and correlation analysis.

Constructs	M ± SD	Sk	Kur	Participation Motivation	Psychological Resilience	Competitive Anxiety
Participation motivation	3.14 ± 0.83	−0.25	−1.17	1		
Psychological resilience	3.25 ± 0.93	−0.36	−1.15	0.64***	1	
Competitive anxiety	2.42 ± 0.76	0.29	−1.48	−0.50***	−0.56***	1

### 3.2 Confirmatory factor analysis

Confirmatory factor analysis was performed in this study for the overall measurement model, which comprised three variables: participation motivation, psychological resilience, and competitive anxiety. The factor loadings exceeded the 0.50 limit, ranging from 0.68 to 0.91. The indices of the measurement model met acceptable levels (Table 3) (Ji et al., 2024).

**Table 3:** Model fit.

Fit index	Reference Value	Model
Chi-square value / Degrees of freedom	<5	1.46
Root mean square error of approximation	<0.08	0.03
Goodness-of-fit index	>0.90	0.92
Adjusted goodness-of-fit index		0.91
Comparative fit index		0.98
Incremental fit index		0.98
Tucker- Lewis index		0.97

### 3.3 Common method bias

Harman’s single-factor test extracted three factors; the first one explained 38.526% of the variation, which was less than the 40% requirement (Podsakoff and Organ, 1986). In addition, we employed the latent variable approach by adding a latent variable to the measurement model and associating it to every observed variable. This model was contrasted with one that did not include the latent variable (Podsakoff et al., 2003). The findings showed that the baseline model had a χ^2^ = 1504.949 (df = 1,031), while the comparison model had a χ^2^ = 1504.949 (df = 1,030), with a difference of 0 (df = 1) (*p* > 0.05). These results imply that common method bias has no appreciable impact on the findings of this study.

### 3.4 Measurement model

The reliability indicators include factor loadings for the items and composite reliability. In general, item factor loadings ought to surpass 0.70. For items with external loadings between 0.40 and 0.70, if removing them significantly improves composite reliability or average variance extracted, these items can be considered for deletion. Based on this, the study removed items with external loadings below 0.70, leading to a significant improvement the validity and reliability of the model. Internal consistency reliability was evaluated using composite reliability, with values expected to exceed 0.70. The composite reliability was found to be satisfactory (see Table 4).

**Table 4:** Reliability and validity.

Constructs	Cronbach’s alpha	Composite reliability	Average variance extracted
Participation motivation	0.939	0.946	0.539
Psychological resilience	0.926	0.938	0.602
Competitive anxiety	0.955	0.959	0.514

Both discriminant and convergent validity were assessed. Convergent validity was evaluated using average variance extracted, and results showed that every average variance extracted score was higher than the 0.50 cutoff. Discriminant validity was tested using the Heterotrait-Monotrait ratio and the traditional Fornell-Larcker criterion. Every Heterotrait-Monotrait ratio (Table 5) was below the 0.90 cutoff. Additionally, all of the construct correlations met the Fornell-Larcker criterion since they were less than the square root of each construct’s average variance extracted value (Table 6). Hence, the findings from both the Heterotrait-Monotrait ratio and Fornell-Larcker approaches demonstrate that the constructs exhibit good discriminant validity.

**Table 5:** Heterotrait-Monotrait ratio criterion.

Constructs	Competitive anxiety	Participation motivation	Psychological resilience
Competitive anxiety			
Participation motivation	0.526		
Psychological resilience	0.594	0.691	

**Table 6:** Fornell-Larcker criterion.

Constructs	Competitive anxiety	Participation motivation	Psychological resilience
Competitive anxiety	***0.717***		
Participation motivation	−0.500	***0.734***	
Psychological resilience	−0.561	0.648	***0.776***

The square roots of the average variance extracted are shown in bold italics on the diagonal.

### 3.5 Structural model

#### 3.5.1 Collinearity test

First, a collinearity test was performed to evaluate the multicollinearity between the latent variables. By empirical standards, the variance inflation factor ought to be less than 3.3. The results (see Table 7) show that the variance inflation factor varied from 1.000 to 1.869, suggesting that model’s multicollinearity is not a major problem.

**Table 7:** Variance inflation factor.

Constructs	Competitive anxiety	Participation motivation	Psychological resilience	Sport commitment
Competitive anxiety				
Participation motivation	1.762		1.000	
Psychological resilience	1.869			
Sport commitment	1.260			

#### 3.5.2 Path hypotheses

**Figure 2:**
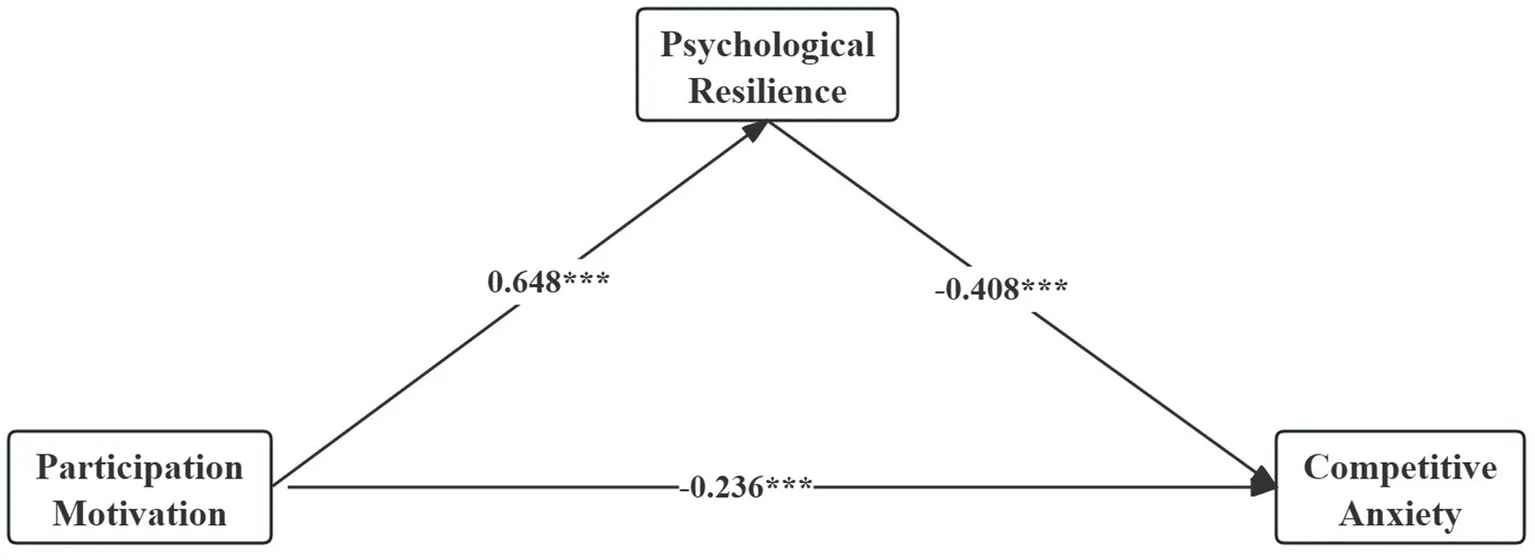
Path diagram.

The findings indicate that participation motivation (*β* = −0.236, *t* = 5.279, *p* < 0.001) is significantly negatively linked with competitive anxiety (Table 8 and Figure 2).

**Table 8:** The mediation models of resilience in the link between participation motivation and competitive anxiety (n = 711).

Path	*β*	*t*	*p*
Participation Motivation → Competitive Anxiety	−0.236	5.279	< 0.001
Participation Motivation → Psychological Resilience	0.648	32.361	< 0.001
Psychological Resilience → Competitive Anxiety	−0.408	9.578	< 0.001

#### 3.5.3 Coefficient of determination (*R*^2^)

*R*^2^ results (Table 9) show that the predictor variables explaining 34.7% of the variance in competitive anxiety. Additionally, all *Q*^2^ are greater than zero (Table 9), indicating the model’s strong predictive value.

**Table 9:** Explanatory power and predictive relevance.

Constructs	*R* ^2^	*Q* ^2^	Model fit
Competitive anxiety	0.347	0.176	SRMR:0.032
Psychological resilience	0.419	0.249	NFI:0.921

### 3.6 Mediation analysis

The findings demonstrated a significant indirect association between participation motivation and competitive anxiety via psychological resilience (*β* = −0.264, *t* = 8.922, *p* < 0.001). After resilience was included in the model, the direct link between participation motivation and competitive anxiety remained significant (*β* = −0.236, *t* = 5.279, *p* < 0.001), indicating partial mediation by psychological resilience. The indirect effect made up 52.8% of the overall effect, suggesting that psychological resilience accounted for a significant amount of the association between participation motivation and competitive anxiety (Table 10).

**Table 10:** Mediation effects of resilience.

Path	*β*	*t*	*p*	Mediation Type
Participation motivation → Psychological resilience → Competitive anxiety	−0.264	8.922	< 0.001	CPM

CPM, complementary partial mediation.

### 3.7 Moderation analysis

This study used the two-stage method to analyze the moderating role of sport commitment in the link between participation motivation and competitive anxiety. The two-stage method is widely used in moderation effect analysis and is regarded as more accurate than the product indicator method and the orthogonal approach (Becker et al., 2018; Henseler and Chin, 2010). Without introducing the interaction term, the *R*^2^ for competitive anxiety was 0.347. After adding the interaction term between participation motivation and sport commitment, the *R*^2^ rose to 0.460, indicating an 11.3% increase in explanatory power. This implies that sport commitment, as a moderating factor, significantly enhances the model. Further analysis revealed a strong negative link between participation motivation and competitive anxiety (*β* = −0.169, *t* = 4.286, *p* < 0.001). The interaction term between participation motivation and sport commitment was also statistically significant (*β* = 0.072, *t* = 2.714, *p* = 0.007) (Table 11). The interaction effect had an effect size of *f*^2^ = 0.009, indicating a small moderating effect of sport commitment. Thus, although the interaction effect was statistically significant, its incremental contribution to the explained variance in competitive anxiety remained limited.

**Table 11:** Moderation analysis.

Path	*β*	*SE*	*t*	*p*
Participation motivation → Competitive anxiety	−0.169	0.039	4.286	<0.001
Sport commitment × Participation motivation → Competitive anxiety	0.072	0.027	2.714	0.007

**Figure 3:**
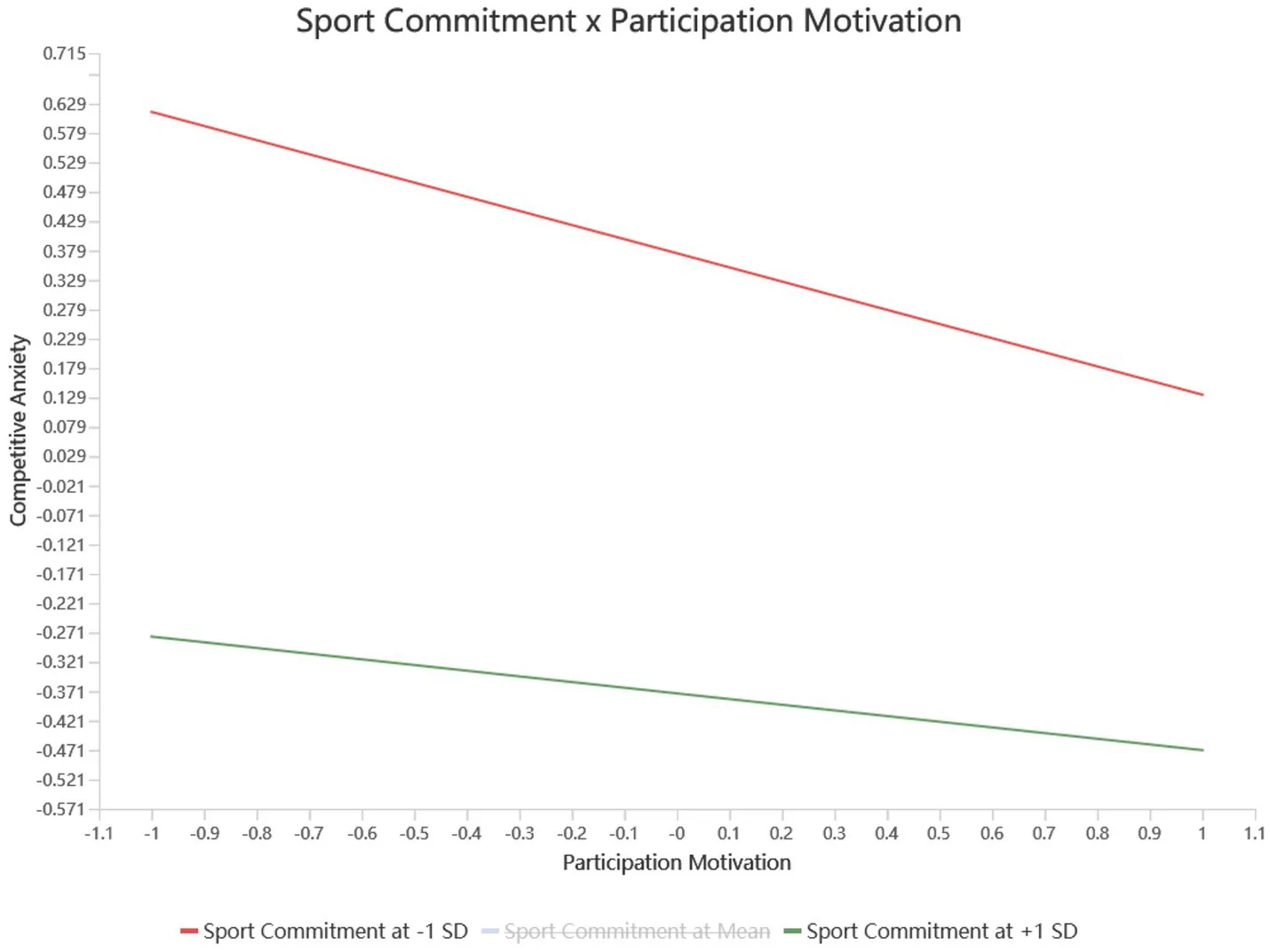
Simple slope analysis of the interaction between participation motivation and sport commitment on competitive anxiety.

To better understand the specific pattern of the moderating impact, this study conducted a simple slope analysis (Figure 3). The findings showed that sport commitment considerably moderated the association between participation motivation and competitive anxiety, although the effect size was small. Participation motivation was negatively associated with competitive anxiety at all levels of sport commitment. However, this negative association gradually weakened as sport commitment increased. The differences among the simple slopes were limited, indicating that sport commitment changed the strength of the association between participation motivation and competitive anxiety only to a small extent. Therefore, although the moderation effect was statistically significant, its practical significance should be interpreted with caution.

## 4 Discussion

The purpose of this study is to investigate the variables associated with competitive anxiety among university student athletes and analyze the mediating role of psychological resilience and the moderating role of sport commitment. The findings indicate a substantial negative link between participation motivation and competitive anxiety, with psychological resilience playing a complementary partial mediating factor between participation motivation and competitive anxiety. Data analysis conducted using Smart-PLS program showed that these constructs together accounted for 34.7% of the variation in competitive anxiety. Sport commitment exhibited a significant moderating effect between participation motivation and competitive anxiety. After adding the interaction term, the variance explanation of competitive anxiety increased by 11.3%, indicating that the moderating effect of sport commitment on this path is statistically significant.

### 4.1 Participation motivation and competitive anxiety

This study identified a significant negative link between participation motivation and competitive anxiety, which supports H1. This result aligns with Haase (2021), that stronger participation motivation can effectively alleviate an individual’s competitive anxiety. Athletes with strong participation motivation typically focus more on the enjoyment of the sport itself and personal growth. This intrinsic participation motivation may be linked to enhanced self-confidence, allowing them to approach the challenges of competitive sports with greater composure, potentially reducing anxiety about competitive failure (Reyes-Hernández et al., 2021). The COR theory states that athletes who focus on intrinsic participation motivation, they are more likely to conserve and replenish psychological resources, which helps them better cope with stress (Hobfoll, 1989). Athletes with strong participation motivation inclined to adopt positive emotional regulation and coping strategies, like meditation, mindfulness, and positive self-feedback, when facing pressure. This active management of emotions may be linked to lower competitive anxiety (Tahir et al., 2024). Therefore, participation motivation provides more avenues for managing stress, which may be associated with reduced competitive anxiety, in line with the COR theory’s resource conservation tenets.

### 4.2 The mediating role of psychological resilience

This study demonstrated that resilience mediates the link between participation motivation and competitive anxiety, confirming H2. This result aligns with Bochaver et al. (2023), suggesting that participation motivation can indirectly alleviate competitive anxiety by enhancing psychological resilience (Tang et al., 2023). Athletes with high participation motivation often view sports as an opportunity for personal growth and challenge, believing in their ability to overcome difficulties. This self-confidence helps them maintain increased psychological resilience when facing obstacles (Alexe et al., 2022). Athletes with great psychological resilience can remain calm under competitive pressure, focusing on the task rather than excessively worrying about competition outcomes or external evaluations. This enhanced ability to cope with stress can be explained by COR theory, with psychological resilience serving as a resource that assist athletes better alleviate competitive anxiety (Wang, 2021). In addition, athletes with higher psychological resilience are able to transform stress into motivation, enhancing their adaptability and better managing their emotions in high-pressure environments, thereby reducing the occurrence of competitive anxiety (González-Hernández et al., 2020). Higher psychological resilience makes athletes more capable of handling competitive pressure. They possess stronger emotional regulation and adaptability, allowing them to maintain composure under strain and reduce competitive anxiety (Salovey et al., 1999). According to the stress appraisal theory, athletes with higher psychological resilience often view pressures as challenges as opposed to dangers. They adopt more proactive coping strategies, which helps reduce anxiety and allows them to focus on the task at hand, rather than excessively worrying about the competition outcome or external evaluations (Tugade and Fredrickson, 2004; Yu et al., 2024). Therefore, athletes with stronger participation motivation typically exhibit higher psychological resilience, which helps them to better control their emotions, reduce competitive anxiety, and maintain consistent performance in competitions.

### 4.3 The moderating role of sport commitment

This research discovered that sport commitment moderates the link between participation motivation and competitive anxiety, supporting H3. This result aligns with Hu et al. (2023), which suggests that a strong commitment driven by passion for the sport can reduce athletes’ fear of failure, thereby alleviating competitive anxiety. Specifically, participation motivation was negatively associated with competitive anxiety, whereas the interaction term was positive. This indicates that the negative association weakened as sport commitment increased. This pattern may suggest that participation motivation and sport commitment have a compensatory relationship in coping with competition pressure. Sport commitment reflects athletes’ relatively stable and sustained engagement in sports participation (Guo et al., 2024; Joung et al., 2023). When sport commitment is high, athletes’ competitive anxiety may vary less across levels of participation motivation. Conversely, when sport commitment is low, the association between participation motivation and competitive anxiety is more pronounced (Tao and Yu, 2025). According to COR theory, different psychological resources may complement one another under stressful conditions. When one resource is relatively abundant, the marginal role of another resource may become weaker (Hobfoll, 1989; Simon-Piqueras et al., 2023). However, the effect size of the interaction was small. This indicates that although the moderating effect of sport commitment was statistically significant, its practical significance should be interpreted with caution.

## 5 Significance and limitations

### 5.1 Theoretical significance

Drawing on COR theory, this study offers a comprehensive exploration of how participation motivation, psychological resilience, and sport commitment are associated with athletes’ competitive anxiety. First, the study concluded a negative link between participation motivation and competitive anxiety, and explored the mediating role of resilience as a psychological resource in this link. By validating the mediating role of resilience between participation motivation and competitive anxiety, the research enhances the use of COR theory in regulating individual emotions and stress under high-pressure environments, particularly in regard to competitive sports. Secondly, the study discovered that sport commitment significantly moderates the link between participation motivation and competitive anxiety, providing new theoretical support for the concept of behavioral resources within COR theory. This further demonstrates that individuals can mitigate the negative impact of external pressure on emotions by strengthening goal-oriented behaviors. This study, by constructing a moderated mediation model, systematically analyzed how athletes utilize psychological and behavioral resources to manage competitive pressure and reduce competitive anxiety. The research offers a new theoretical framework for illuminating the complex interactions between participation motivation, psychological resilience, and sport commitment in reducing competitive anxiety, offering a theoretical foundation for the creation of psychological intervention strategies for athletes. Overall, this study extends the use of the COR theory in competitive sports and offers a fresh theoretical viewpoint for psychological interventions for athletes. Specifically, it highlights the importance of enhancing psychological resilience and sport commitment, offering valuable theoretical support for improving athletes’ mental health and athletic performance.

### 5.2 Practical significance

Coaches can enhance athletes’ sense of involvement by stimulating their participation motivation, thereby effectively reducing competitive anxiety. Specifically, coaches can provide personalized feedback or involve athletes in the decision-making process for training and competitions to increase their feeling of autonomy and control, thus increasing their participation in training. At the same time, coaches should set challenging yet achievable goals and use goal-oriented motivational strategies to enhance athletes’ sense of accomplishment and reduce anxiety. In addition, coaches should avoid over-relying on external reward systems. While external rewards can provide short-term motivation, they may weaken intrinsic motivation and reduce sport commitment in the long run. Over-dependence on external rewards may lead athletes to focus too much on the rewards, neglecting the intrinsic value of the sport. Therefore, coaches should place greater emphasis on enhancing athletes’ intrinsic motivation, providing positive feedback, and offering emotional support to foster their passion and self-drive, thereby improving their sport commitment and long-term performance.

For sports psychologists, this study emphasizes the key role of psychological resilience in alleviating competitive anxiety. Sports psychologists can design specialized resilience training programs, such as scenario simulations, deep breathing exercises, and mindfulness meditation techniques, to help athletes enhance their emotional regulation and stress coping abilities in high-pressure environments. Additionally, regularly conducting stress management workshops to teach athletes effective coping strategies is also an effective way to reduce anxiety.

For athletes, this study provides specific self-regulation strategies to enhance participation motivation and sport commitment during training and competitions. Athletes can boost their participation motivation by setting small and specific training goals and strengthen their sport commitment through daily self-motivation and goal reflection, helping them stay focused and stable under pressure. Additionally, athletes should regularly practice emotional regulation practices like meditation and deep breathing, to enhance psychological resilience and reduce emotional fluctuations and anxiety levels.

In summary, this study provides specific and effective psychological intervention strategies for coaches, sports psychologists, and athletes. These strategies not only help athletes alleviate competitive anxiety but also enhance their psychological adaptability, thereby improving athletic performance and long-term development.

### 5.3 Limitations and future research directions

First, although the cross-sectional design of this study finds links between variables, it is unable to demonstrate causal linkages. Therefore, future research should use longitudinal designs or experimental interventions to more accurately verify the causal relationships between participation motivation, psychological resilience, and sport commitment, as well as their long-term effects on competitive anxiety. Second, only Chinese university student athletes were included in the sample. The specific cultural and educational context may restrict the applicability of the findings to other athlete populations. Future research should include athletes from different countries, cultural backgrounds, and competitive levels to further examine the cross-cultural stability and applicability of the findings. Additionally, this study did not examine in depth the relationship between various aspects of participation motivation and competitive anxiety. Future research could refine the motivational dimensions and test the role of different types of motivation in competitive anxiety, further enriching the theoretical framework. Besides, the model constructed in this study is static and does not fully consider the dynamic process of athletes’ psychological changes. Future research could use dynamic models to explore how athletes regulate competitive anxiety over time through factors such as participation motivation, psychological resilience, and sport commitment, providing more practically meaningful guidance for intervention strategies. Finally, this study primarily focuses on state competitive anxiety. Future research should examine the process through which state competitive anxiety transitions into trait competitive anxiety, to learn more about how anxiety develops over time. Additionally, future studies could separately investigate somatic anxiety and cognitive anxiety, providing a more comprehensive analysis that helps better understand the specific impacts of different types of anxiety on athlete performance.

## 6 Conclusion

Based on COR theory, this study investigated the link between participation motivation and competitive anxiety among university student athletes, emphasizing the mediating role of resilience and the moderating role of sport commitment. The findings demonstrated a significant negative link between participation motivation and competitive anxiety, with psychological resilience playing a significant mediating role. Additionally, sport commitment played an important moderating role in the link between participation motivation and competitive anxiety. Theoretically, this research expands our knowledge of the processes by which participation motivation, psychological resilience, and sport commitment are associated with competitive anxiety. Practically, the research provides valuable theoretical basis for creating psychological intervention strategies for university student athletes, particularly in enhancing participation motivation, sport commitment and psychological resilience. A limitation of the study is that the sample was limited to university student athletes in China. Future research could expand the sample to increase the findings’ generalizability.

## Data Availability

The raw data supporting the conclusions of this article will be made available by the authors, without undue reservation.

## Footnotes

1
https://www.wjx.cn/
